# The use of alfaxalone for short-term anesthesia can confound serum progesterone measurements in the common marmoset: a case report

**DOI:** 10.5194/pb-9-23-2022

**Published:** 2022-07-27

**Authors:** Maria Daskalaki, Charis Drummer, Rüdiger Behr, Michael Heistermann

**Affiliations:** 1 Platform Degenerative Diseases, German Primate Center – Leibniz Institute for Primate Research, Kellnerweg 4, 37077 Göttingen, Germany; 2 Endocrinology Laboratory, German Primate Center – Leibniz Institute for Primate Research, Kellnerweg 4, 37077 Göttingen, Germany

## Abstract

Alfaxan^®^ (alfaxalone) is a steroid general anesthetic widely used in veterinary medicine for induction and maintenance of anesthesia in several species. While the use of alfaxalone in veterinary practice has several benefits compared to the use of other anesthetic agents, the fact that it is derived from progesterone may confound the measurement of the latter in the blood of animals under alfaxalone treatment. In the present case study, we report the measurement of serum progesterone in an individual common marmoset (*Callithrix jacchus*) during five ovarian cycles in which luteolysis was induced by PGF2 α . Blood samples were usually taken from the awake animal with the exception of the fifth cycle in which the sample was collected under alfaxalone anesthesia in connection with a tooth extraction. In contrast to the previous four cycles in which luteolysis resulted in the expected marked decrease in progesterone concentrations, the – apparent – progesterone level in the cycle under alfaxalone treatment remained unexpectedly high. Cross-reactivity of the non-specific antibody used in the progesterone assay with alfaxalone most likely explains this finding.

## 1 Introduction

Alfaxalone is a veterinary general anesthetic which enhances the inhibitory effects of γ -aminobutyric acid (GABA) on GABA ${}_{A}$ receptors, causing nerve cell hyperpolarization and blocking neural impulse transmission (Ghit et al., 2021; Papich, 2016; Albertson et al., 1992). It shows certain benefits compared to other anesthetic agents due to a broad margin of safety and a low cumulative effect after repeated doses, which enables a rapid recovery (Lau et al., 2013; Ferre et al., 2006; Rezende, 2015). Alfaxalone is used for both induction and maintenance of anesthesia in many species including companion and experimental animal species such as dog (White and Yates, 2017), cat (Khenissi et al., 2017), goat (Abouelfetouh et al., 2021), sheep (Andaluz et al., 2012), pig (Bigby et al., 2017), horse (Goodwin et al., 2011), rhesus macaque (Bertrand et al., 2017), cynomolgus monkey (Casoni et al., 2015), and common marmoset (Bakker et al., 2013). Alfaxalone anesthesia has minimal negative impact on the cardiorespiratory system though it can cause hypoxemia if administrated in high doses (Wada et al., 2020). It is also commonly used without side effects in pregnant dogs showing no negative effects on the stability of maternal and fetal hemodynamics (Andaluz et al., 2013; Metcalfe et al., 2014). Altogether, alfaxalone is regarded as a safe anesthetic agent and is widely used in high-risk patients (Bosing et al., 2012). However, it has a very poor analgesic effect (Bennell et al., 2019).

**Figure 1:**
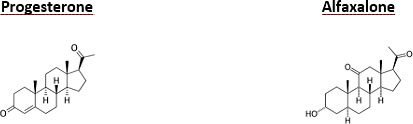
Chemical structure of progesterone and alfaxalone.

Chemically, alfaxalone is a synthetic pregnane steroid, namely 3 α -hydroxy-5 α -pregnan-11,20-dion (Fig. 1). It is a derivative of progesterone and allopregnanolone, differing from the latter only by the addition of a ketone group at C11, though it has no glucocorticoid or mineralocorticoid action (Ferre et al., 2006; Rezende, 2015).

While the use of alfaxalone in veterinary practice has several advantages compared to the use of other anesthetic agents as mentioned above, the fact that it is derived from progesterone and is structurally similar to naturally occurring pregnanediones and pregnanolones suggests that it may confound the measurement of progesterone in the blood of animals under alfaxalone treatment. This is due to the fact that antibodies used for the quantification of progesterone by immunological methods (e.g., ELISA, RIA) often cross-react with 5 α - or 5ß-reduced progestogens (Graham et al., 2001) to which alfaxalone belongs. A co-measurement of alfaxalone in serum progesterone assays might therefore be possible (as also suggested by findings in cats; Trumble et al., 2020), even if the degree of cross-reactivity with the progesterone antibody may be low. Here, we report a case of an unusually high serum progesterone value in a female common marmoset anesthetized by intramuscular injection of alfaxalone during the follicular phase of its ovarian cycle.

Common marmosets (*Callithrix jacchus*) are widely used non-human primates in biomedical research and translational medicine (Kishi et al., 2014). Studies conducted in these areas often require a detailed monitoring of female reproductive condition, including information on ovarian activity and the exact state of the ovarian cycle (Drummer et al., 2021). Such profound knowledge of the reproductive cycle is required for instance to monitor hormonal stimulation or to determine the optimal time points for oocyte or embryo retrieval and embryo transfer (Drummer et al., 2021; Marshall et al., 2003). Determining the female reproductive status, including ovarian cycle stage, can be achieved through measurements of progesterone in small blood samples which are usually collected from awake animals (Saltzman et al., 1997). Based on a threshold value of 10 ng mL ${}^{-1}$ progesterone indicating occurrence of ovulation in the marmoset, the follicular and luteal stages of the ovarian cycle can be reliably determined (Harlow et al., 1983). Thus, any confound in the progesterone measurement could lead to a false interpretation of a female's cycle stage, and in this respect, caution might be advised for serum progesterone determination in samples collected under alfaxalone treatment (Trumble et al., 2020). Here we describe a case study of an unexpectedly elevated progesterone value with the blood sample taken under alfaxalone anesthesia in a female common marmoset. Since alfaxalone is structurally very similar to allopregnanolone (see above), which shows a 64 % cross-reactivity in our progesterone assay (Graham et al., 2001), we assessed the probability that interference of alfaxalone in our progesterone assay due to potential cross-reactivity was responsible for the unexpectedly high progesterone value recorded.

## 2 Materials and methods

### 2.1 Animal

​​​​​​​We describe the case of a 4-year-old female common marmoset (animal number no. 17402) enrolled as an oocyte donor in an experiment aiming at the generation of genetically modified animals. The animal was obtained from the breeding colony of the German Primate Center and the experimental procedures were authorized by the federal authorities (LAVES) under license number 33.19-42502-04-19/3221. Health and general condition of the animal were controlled daily by experienced animal care takers and regularly by veterinarians. The animal was pair-housed with an intact male and was fed a marmoset-specific diet, supplemented with fruits and vegetables as described elsewhere (Drummer et al., 2021). Water was available ad libitum.

**Figure 2:**
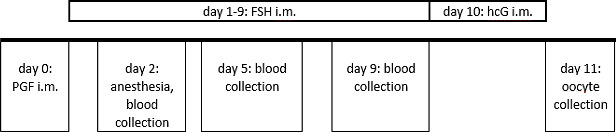
Ovarian stimulation and blood sampling protocol in the reported case. Routinely, blood collection is performed on day 2 on the non-anesthetized animal.

The animal's ovarian cycle was routinely monitored twice a week by blood collection from the femoral vein without anesthesia in an awake condition, to determine progesterone concentration. In the mid-luteal phase synthetic prostaglandin F2 α (PGF2 α ; 0.2 mL of a mixture of 0.1 mL Estrumate^®^ 250 µ g mL ${}^{-1}$ , Intervet Deutschland GmbH, Unterschleißheim, Germany, and 3.2 mL Ringer lactate solution, B. Braun SE, Melsungen, Germany) was injected into the femoral musculature in order to induce luteolysis and thus to initiate a new follicular phase (Summers et al., 1985). The day after PGF2 α administration and the 8 following days, the animal was given 25 IU FSH (follicle-stimulating hormone, GONAL-f^®^ 450 IU/0.75 mL, Merck Europe B.V., Amsterdam, the Netherlands) intramuscularly (femoral musculature) to stimulate the ovaries. On the second day of FSH application, we detected a brownish, broken canine in the right upper jaw and decided to remove the tooth. The animal was anesthetized with a combination of diazepam (0.05 mL per animal, Diazepam-ratiopharm^®^ 10 mg/2 mL, Ratiopharm GmbH, Ulm, Germany) and alfaxalone (0.1 mL per 100 g body weight, 0.4 mL in total, Alfaxan^®^ 10 mg mL ${}^{-1}$ , Jurox, Dublin, Ireland), blood for routine progesterone measurement was collected under anesthesia, and the canine was removed afterwards. Appropriate analgesics and antibiotics were applied. The animal recovered from anesthesia without any complications. Three and 7 days later (i.e., on days 5 and 9 post PGF2 α administration) additional blood samples were collected for progesterone monitoring with the animal being awake and non-sedated (Fig. 2).

### 2.2 Progesterone measurement and alfaxalone cross-reactivity

**Figure 3:**
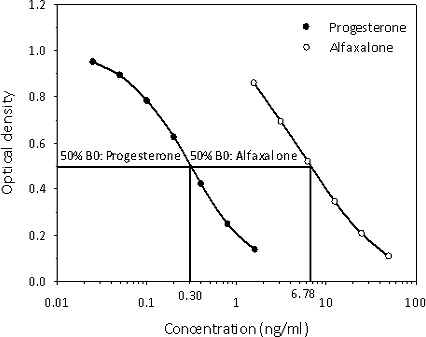
Cross-reactivity determination of alfaxalone measurement in the progesterone enzyme immunoassay used for analysis of serum progesterone concentrations of common marmosets. Concentration values at 50 % binding (50 % B0) for progesterone and alfaxalone used for calculation of the alfaxalone cross-reactivity data are indicated.

Progesterone was determined in the blood serum by a direct, non-extraction enzyme immunoassay (EIA) using a monoclonal antibody (Quidel clone no. 425; CL425) produced against 11-hydroxyprogesterone-hemisuccinate: BSA (Grieger et al., 1990), and progesterone–horseradish peroxidase (HRP) was used as a conjugate. Peripheral plasma progesterone measurement is routinely used for ovarian cycle monitoring in our female common marmosets (Harlow et al., 1983). The assay has been analytically validated by demonstrating (i) high sensitivity (90 % binding = 50 pg mL ${}^{-1}$ ), (ii) parallelism between displacement curves from standards and dilutions of plasma, and (iii) precision (intra- and interassay CVs of high and low value quality controls = < 10 % and < 15 %, respectively). Moreover, the assay produces biologically valid results by demonstrating the reliable discrimination of ovarian cycle stages in the marmoset, with progesterone concentrations < 10 and > 10 ng mL ${}^{-1}$ indicating the follicular and luteal phase of the ovarian cycle, respectively (Harlow et al., 1983) (see Fig. 3 as example).

The assay's antibody has a high cross-reactivity with progesterone but also substantially cross-reacts with other pregnanediones as well as with pregnanolones (Graham et al., 2001). Due to its non-specificity, the EIA has been widely used for monitoring progesterone and progesterone metabolites in blood and feces, respectively, of a variety of species (Graham et al., 2001).

Since we assumed that alfaxalone would interfere in the measurement of serum progesterone (see Introduction), we determined its degree of cross-reactivity in our progesterone assay and found it to be 4.4 % (Fig. 3).

## 3 Results

**Figure 4:**
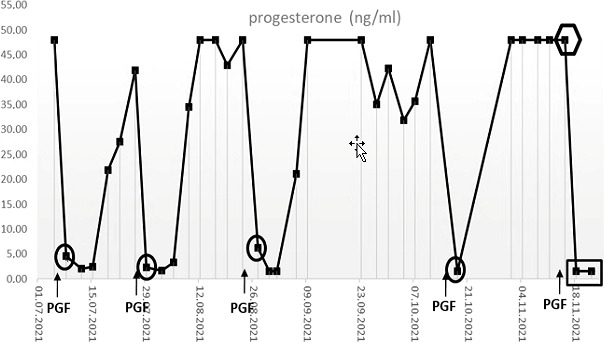
Serum progesterone levels in the animal #17402 after the intramuscular injections of PGF2 α (PGF) during several ovarian cycles. The sample highlighted by the hexagon was taken under alfaxalone anesthesia. Note that this sample was taken 2 d after PGF2 α application, which caused the expected marked decline in progesterone concentrations in the previous cycles.

Under normal conditions (i.e., when blood was collected in a non-sedated state), the animal reacted to the application of the PGF2 α with the expected decline of progesterone concentrations to ≤ 10 ng mL ${}^{-1}$ within 1–2 d, indicative of the onset of a new follicular phase (Fig. 4, circles) (Summers et al., 1985). In the sample collected under alfaxalone anesthesia 2 d after induction of luteolysis, however, the determined (apparent) serum progesterone concentration was not decreased but stayed elevated (Fig. 4, hexagon), suggesting that the animal might not have responded to the PGF2 α administration. However, in the samples collected on days 5 and 9 after PGF2 α injection progesterone concentrations were < 10 ng mL ${}^{-1}$ (Fig. 4, rectangle), indicating a successful luteolysis and showing that the animal had indeed responded properly to the PGF2 α treatment like in previous cycles. The markedly elevated progesterone value on day 2 post PGF2 α treatment was therefore most likely related to the alfaxalone anesthesia, which we discuss in detail in the following.

## 4 Discussion

Alfaxan^®^ (alfaxalone) is an anesthetic agent widely used in veterinary medicine for induction and immersion of anesthesia (Rezende, 2015). We frequently use alfaxalone in marmosets for short-term anesthesia as it is well tolerated, safe and the animals wake up fast and gently (Bakker et al., 2013). The common marmoset (*Callithrix jacchus*) is a useful animal model in translational medicine (Kishi et al., 2014). One big advantage of this model is the small size of the animals, which facilitates handling, including blood sample collection for hormone measurements and other blood chemistry diagnostics, without anesthesia (Saltzman et al., 1997). The case we describe here refers to an animal enrolled in an experiment for the generation of transgenic marmosets. For this purpose, we monitored the ovarian cycle by measuring the blood progesterone levels twice a week. In contrast to previous cycles in which blood was collected without anesthesia, we found an unexpectedly elevated progesterone value following luteolysis in the sample collected under anesthesia. We speculated that alfaxalone might have confounded the measurement of native progesterone in this sample, particularly because our progesterone antibody substantially cross-reacts with 5 α -reduced pregnanediones and pregnanolones (Graham et al., 2001) to which, structurally, alfaxalone belongs. We determined a cross-reactivity for alfaxalone of 4.4 % in our progesterone EIA, a relatively low value. However, because the dosage of alfaxalone required for sedation is rather high (1 mg per 100 g body weight; 4 mg in total for the study animal), we calculated that even if only 20 % of the injected alfaxalone was present in the circulation during the time when the blood sample was collected, our progesterone immunoreactivity value would have been raised by about 30 ng mL ${}^{-1}$ . Thus, co-measurement of alfaxalone in our progesterone EIA was substantial and significantly interfered with the immunological determination of blood progesterone levels (Trumble et al., 2020). This was indirectly confirmed by the finding that in the samples collected on days 5 and 9 after PGF2 α injection when the animal was not sedated, the progesterone concentrations showed the expected low values, indicating that the animal had indeed responded properly to the PGF2 α treatment like in previous cycles. Since alfaxalone, despite being a progesterone derivative, does not bind to glucocorticoid, mineralocorticoid or sex hormone receptors (Ferre et al., 2006), we assume that the observed interference was not due to an increased alfaxalone-induced progesterone production.

Given that pregnanediones and pregnanolones are abundant metabolites of progesterone in the urine and feces of many animal species (Graham et al., 2001) and considering that alfaxalone metabolism takes place mainly in the liver but also in the kidney and lung (Ferre et al., 2006), we envisage that the measurement of urinary/fecal progestogens in non-invasive samples collected from animals which had received alfaxalone anaesthesia may also be confounded by excreted alfaxalone metabolites. This needs to be confirmed though.

Generally, the use of a highly specific progesterone antibody should substantially reduce or even overcome the problem of co-measurement of alfaxalone. To estimate the potential risk of a confounding co-measurement as encountered by us, we recommend to determine the alfaxalone cross-reactivity in the progesterone assay of choice prior to the collection of blood samples under alfaxalone anesthesia.

As alfaxalone does not interact with the hormonal steroid receptors (Visser et al., 2002) we do not expect any progesterone-like biological function of the anesthetic agent and therefore no unfavorable impact on the female reproductive system. Even if there is a biological effect of alfaxalone, this would be presumably only short-term given that the half-life of the compound is relatively short. Mean terminal plasma half-life in the recommended clinical dose is about 24 min in dogs (Ferre et al., 2006) and 45 min in cats (Whittem et al., 2008) after a single i.v. application of 2 and 5 mg kg ${}^{-1}$ respectively.

Progesterone measurement in blood serum is a routine analysis in veterinary reproduction medicine (Brugger et al., 2011; Saltzman et al., 1997; Hammer and Howland, 1991). Mostly, blood collection procedures are well tolerated by animals, at least domesticated and/or small-bodied ones and those habituated to the procedure (Saltzman et al., 1997). In larger-bodied non-domesticated species and in cases of uncooperative or aggressive animals, sedation is required prior to the blood-taking procedure (Hotchkiss and Young, 2020). Our present finding shows that in such cases attention should be paid on the selection of the anesthetic agent when the blood sampling is also undertaken for progestogen measurements. Under such conditions the use of alfaxalone as the anesthetic agent should be avoided as it may confound the progesterone determination. If unnoticed, this may lead to false interpretations regarding progesterone values with potentially far-reaching consequences, such as break-off of artificial reproduction technologies, like ovarian stimulation treatments in animals selected for oocyte donor studies. In the case of a concomitant veterinary intervention in anesthesia, it is preferable to do all the procedures in the anesthetized animal as this minimizes the stress for the monkeys and is consistent with animal welfare. If progesterone determination is required from animals under anesthesia, the combined application of xylazine and ketamine or medetomidine and ketamine could be an adequate alternative to alfaxalone (Goodroe et al., 2021).

We conclude that alfaxalone can confound immunoassay-based progesterone determination and should be avoided for anesthesia prior to blood sampling, when the progesterone blood level has to be determined.

## Data Availability

All raw data can be provided by the corresponding authors upon request.
